# Small and Large Animal Veterinarian Perceptions of Antimicrobial Use Metrics for Hospital-Based Stewardship in the United States

**DOI:** 10.3389/fvets.2020.00582

**Published:** 2020-09-08

**Authors:** Laurel E. Redding, Brandi M. Muller, Julia E. Szymczak

**Affiliations:** ^1^Department of Clinical Sciences, University of Pennsylvania School of Veterinary Medicine, Philadelphia, PA, United States; ^2^Department of Biostatistics, Epidemiology and Informatics, University of Pennsylvania Perelman School of Medicine, Philadelphia, PA, United States

**Keywords:** veterinarian, antimicrobial use, metric, antimicrobial stewardship, feedback

## Abstract

**Background:** Robust measurement and tracking of antimicrobial use (AMU) is a fundamental component of stewardship interventions. Feeding back AMU metrics to individual clinicians is a common approach to changing prescribing behavior. Metrics must be meaningful and comprehensible to clinicians. Little is known about how veterinary clinicians working in the United States (US) hospital setting think about AMU metrics for antimicrobial stewardship.

**Objective:** To identify hospital-based veterinary clinicians' attitudes toward audit and feedback of AMU metrics, their perceptions of different AMU metrics, and their response to receiving an individualized prescribing report.

**Methods:** Semi-structured interviews were conducted with veterinarians working at two hospitals in the Eastern US. Interviews elicited perceptions of antimicrobial stewardship in veterinary medicine. Respondents were shown a personalized AMU Report characterizing their prescribing patterns relative to their peers and were asked to respond. Interviews were recorded, transcribed, and analyzed using the framework method with matrices.

**Results:** Semi-structured interviews were conducted with 34 veterinary clinicians (22 small animal and 12 large animal). Respondents generally felt positive about the reports and were interested in seeing how their prescribing compared to that of their peers. Many respondents expressed doubt that the reports accurately captured the complexities of their prescribing decisions and found metrics associated with animal daily doses (ADDs) confusing. Only 13 (38.2%) respondents felt the reports would change how they used antimicrobials. When asked how the impact of the reports could be optimized, respondents recommended providing a more detailed explanation of how the AMU metrics were derived, education prior to report roll-out, guidance on how to interpret the metrics, and development of meaningful benchmarks for goal-setting.

**Conclusions:** These findings provide important insight that can be used to design veterinary-specific AMU metrics as part of a stewardship intervention that are meaningful to clinicians and more likely to promote judicious prescribing.

## Introduction

Antimicrobial stewardship has been defined as “coordinated interventions designed to improve and measure the appropriate use of [antimicrobial] agents by promoting the selection of the optimal [antimicrobial] drug regimen including dosing, duration of therapy, and route of administration” (1). In people, antimicrobial stewardship programs (ASPs) have been shown to improve patient outcomes, shorten the length of stay, reduce antimicrobial resistance, and save money in the inpatient setting (2).

A frequently used stewardship initiative is the provision of periodic feedback on a prescriber's AMU, oftentimes situating their use relative to their peers'. In human medicine, this type of intervention has been shown to decrease AMU, improve clinical outcomes, and decrease costs in the outpatient clinical setting (3–6). In animal agriculture, AMU is tracked at the farm level in many (mostly European) countries, and individual AMU data are regularly provided to producers and veterinarians for purposes of benchmarking in the context of regulatory programs or quality assurance schemes. These types of initiatives are thought to be major contributors to the decline in AMU observed in many livestock sectors in these countries (7–9).

In veterinary hospitals, AMU data are rarely tracked and much less frequently fed back to clinicians. Veterinary hospitals represent fundamentally different prescribing ecosystems than farms, and very little is known about the attitudes of veterinary clinicians working in these settings toward antimicrobial stewardship initiatives involving tracking and reporting of antimicrobial use, especially in the United States (US). With increasing interest in antimicrobial stewardship within US veterinary hospitals (10, 11), more information is needed on best practices for implementing systems that involve the feedback of AMU prescribing data to veterinarians. In particular, there is no consensus on which metric(s) to use. In farm-level benchmarking schemes, a variety of metrics are used, including count-based, mass-based, daily dose-based, and course-based indicators (12). Each metric has advantages and disadvantages and may or may not be applicable to the hospital setting, where individual animals rather than herds are treated.

Because the goal of providing AMU prescribing data to clinicians is to effect behavior change related to antimicrobial prescribing, the best metrics to use will ultimately be those that are understood, accepted by individual clinicians, and make sense for the context in which they work (13). Feedback of AMU data to human medicine clinicians has mostly been successful in outpatient primary care settings (3–6). It has been less successful in improving the behavior of hospital-based clinicians (14). This may partially be explained by the fact that the social context of work in hospitals is different than in outpatient offices, where multiple clinicians may care for the same patient and responsibility for a prescription is not clearly linked to an individual clinician (15, 16). Sociobehavioral research on the way human medicine clinicians respond to AMU data targeted at them individually demonstrates that considerable skepticism and lack of trust can surround feedback reports, contributing to gaming and workarounds (17). Before implementing an ASP intervention, clinician confidence in the measurement system must be secured to boost clinicians' acceptance of feedback data and increase motivation to change (18, 19). The goal of this study was therefore to identify the perceptions that veterinary clinicians working in the US hospital setting hold toward different AMU metrics used for the systematic tracking and reporting of AMU for purposes of stewardship.

## Methods

### Study Design, Setting, and Participants

We conducted in-depth, semi-structured interviews with a purposive sample of veterinary clinicians from two hospitals (one large animal and one small animal) within a health system in the Eastern US. The small animal hospital sees ~35,000 patients per year and can house 250 inpatients at any time. The large animal hospital sees 4,900 patients per year and can house 200 inpatients at any time. The health system in which we gathered data did not have a formal stewardship program in place that utilized audit and feedback of AMU metrics at the time of data collection. However, both hospitals had implemented individual antimicrobial stewardship initiatives, had held educational sessions on AMU in veterinary medicine, and emphasized that improving the use of antimicrobials was an institutional priority.

This qualitative study was led by a medical sociologist (JES) with expertise in mixed-methods research on antimicrobial prescribing and stewardship interventions, in collaboration with a veterinary epidemiologist (LER) with expertise in developing novel stewardship measures and metrics in veterinary medicine. At each hospital, we sought to interview veterinary clinicians from different specialties who commonly prescribed antimicrobials including internal medicine, surgery, dermatology, and emergency medicine. To identify respondents, we worked with key contacts at each hospital to identify the names and email address of eligible clinicians. The study team recruited respondents by email. Respondents were offered a $50 Visa gift card as an incentive. Potential respondents were assured that their specific comments would not be shared with key contacts beyond a report of de-identified aggregated themes. Our protocol was approved by the University of Pennsylvania Institutional Review Board (IRB Protocol # 832630).

### Data Collection

Data were gathered from April to July 2019. Interviews were conducted in person by the medical sociologist and a senior research associate (BMM) with graduate training in anthropology and advanced interview technique. A semi-structured interview guide was created based on a review of the literature and the authors' previous research (see Supplementary Material for the guide). Questions were designed to be open-ended in order to elicit in-depth responses from veterinarians with minimal prompting by the interviewer (20). Key thematic domains in the interview guide included respondent perceptions of antimicrobial resistance and overuse in veterinary medicine, the application of principles of antimicrobial stewardship to the veterinary hospital context, and perceptions of a personalized Antimicrobial Use Report (described in more detail below). All interviews were, with permission, recorded. Respondents were made aware that the purpose of the study was to better understand their opinions and perceptions of antimicrobial stewardship interventions and AMU metrics in veterinary medicine in order to inform the development of future health system interventions. Interviewers kept ongoing data collection memos to monitor for identification of novel insights and saturation of key themes in order to determine sample size adequacy (21).

#### Personalized Antimicrobial Use Report

The last part of the interview involved presenting the respondent with a hard copy of a personalized Antimicrobial Use Report (see Supplementary Material for sample report). These reports were generated from our veterinary hospital administrative and electronic medical record databases as previously described (22). Briefly, individuals' antimicrobial prescribing patterns from 2013 to 2018 were characterized relative to their peers using metrics that are frequently used to characterize AMU, including count- and dose-based metrics involving the animal-defined daily dose [ADD—also known as the DDDvet (12)], a metric that represents the average maintenance dose of a drug for its main indication in a specified species (12, 23). Standard doses used for the calculation of the ADDs (i.e., the defined daily dose) were obtained either from the drug labels or based on convention in our hospitals. Specifically, the report provided data on (1) the percent of visits in which an antimicrobial or highest priority critically important antimicrobial (HP-CIA—an antimicrobial class that is the sole, or one of limited available therapies, to treat serious bacterial infections in people) (24) was prescribed; (2) the prescription rate, or number of antimicrobial ADDs per 1,000 patient-days; (3) the average number of antimicrobial ADDs per patient; (4) the average number of antimicrobial classes prescribed per visit; (5) and rankings of the most frequently prescribed classes and combinations of antimicrobials. Data were presented via both prose and graphics, and detailed definitions of the ADD metrics along with examples of its use were provided (see example report).

For the large animal hospital clinicians, prescribing patterns were situated relative to peers within their service. Because some of the small animal hospital clinicians were involved in multiple services, their prescribing patterns were situated relative to peers within the entire hospital rather than within their service. For clinicians for whom there were insufficient prescribing data to create a personalized report (e.g., clinicians who were not employed by the hospitals from 2013 to 2018), a mock report was generated using data from a randomly selected de-identified clinician who was present during the time period of interest. These clinicians were made aware when presented the report that it did not contain their actual data, and they were asked to imagine how it might feel to receive such a report. The interviewer gave the respondent as much time as they needed to review the report and then asked them a series of questions to elicit their perceptions about the report and each of the metrics.

### Data Analysis

All audio files were transcribed and uploaded into NVivo 12 software for coding (25). Data were independently analyzed using a flexible coding approach by two coders (26). Themes were systematically identified in a two-stage process. First, a codebook based on the interview guides and a review of the data collection memos was developed. Codes were defined clearly and discussed among the team. Second, the coders applied the codebook to the transcripts. Intercoder reliability was monitored throughout, and modifications were made to the coding procedure to ensure agreement exceeded 95%. Once line-by-line coding was complete, we utilized a framework matrix to identify variation in patterns across codes, respondent classifications, and hospitals (27).

## Results

### Characteristics of Study Subjects

Interviews were conducted with 34 veterinarians. The majority of respondents worked in a small animal hospital setting and had been in practice 10 years or less (Table 1). Interviews ranged in length from 18 to 68 min, with a median of 32 min. Twenty-one (61.8%) respondents were shown their actual prescribing data while 13 (38.2%) were shown mock prescribing data.

**Table 1:** Characteristics of interview respondents.

**Respondent Characteristic (*n =* 34)**	**No. (%) Respondents**
Hospital	
Small animal	22 (64.7)
Large animal	12 (35.3)
Professional role
Faculty	11 (32.4)
Resident	23 (67.6)
Primary specialty	
Dermatology	4 (11.8)
Emergency medicine	4 (11.8)
Internal medicine	15 (44.1)
Oncology	1 (2.9)
Surgery	10 (29.4)
Years in practice	
0–3	12 (35.3)
4–10	12 (35.3)
11–20	6 (17.7)
21–30	3 (8.8)
31+	1 (2.9)

### Initial Impression of Report

Upon initial presentation, 8 (23.5%) respondents expressed negative feelings about the report, 13 (38.2%) expressed positive feelings, while 13 (38.2%) were neutral in their response. Of those respondents who were shown their actual prescribing data, 6 (28.5%) expressed negative feelings about the report while 11 (52.3%) expressed positive feelings about the report. The primary reason respondents gave for feeling negatively about the report was believing that their actual antimicrobial use performance was better than the report indicated (Table 2, Quote 1 [Q1]). The surprise at seeing one's poorer than expected performance coupled with comparison to colleagues led some respondents to explain that the report made them feel “judged” (Q2). Respondents who were pleased explained that their data was on par with or better than how they perceived their actual use of antimicrobials (Q3).

**Table 2:** Interview themes and exemplar quotations.

**Initial Impression of the Report**	
Negative feeling about report	**Q1**. I hate to say that I don't know that this would change what I do. It's definitely food for thought, though, seeing such a high number, because I would sit here and say I'm very cognizant of antimicrobial overprescribing and stewardship, and then I look here and see mine. It's so much worse than I was expecting, but if I do think about it, pretty much every patient I cut gets—not necessarily to go home—will get an injection of antimicrobials. -*Small Animal Surgical Resident* **Q2**. I'm trying to understand those metrics. Yeah. Well, I will say on kind of first looking through it, I felt like I prescribed a lot of antimicrobials. I think I felt an initial sense of being judged. -*Small Animal Internal Medicine Resident*
Positive feeling about report	**Q3**. Initial impression of the package is I'm proud of myself. It's kind of fun to see how you compare to other people in the population. I think I'm kinda on the lower end of prescribing; I tend to not prescribe more than one antimicrobial at once, and I tend not to prescribe it for more than a week, if I'm reading this correctly. -*Small Animal Internal Medicine Faculty*
Report useful to raise awareness	**Q4**. Yeah, I think sometimes you may not realize how much we're actually prescribing or what we're doing because you're just thinking of this case, right in front of me. So to see this listed out on the chart like “oh my God, do all my cases really need these things?” -*Large Animal Internal Medicine Faculty*
Metrics in report cannot account for nuances of prescribing	**Q5**. I think a hard thing to factor into this is sometimes when we're prescribing a drug we might—if a patient, let's say, has osteomyelitis and requires a long treatment with antimicrobial, even in human medicine, I might prescribe all 6 weeks of the antimicrobial at that time, which will markedly increase some of these values. Or I might give them only a 1 or 2-week course, and then recommend they recheck, and then we'll prescribe again at that point. But it might be me prescribing again; at another point, it might be one of my colleagues, or it might be one of those primary—or it might be the patient's primary veterinarian. -*Small Animal Emergency and Critical Care Resident*
Concern about fair comparisons	**Q6**. I mean, I would feel fine about it personally. I feel like everybody can do better and so there's really no harm in that comparison. I think the only challenging thing would be how do you decide who people are being compared to. Like is it hospital-wide? Is it within a department? Is it residents only? Is it interns only? Because I think that there is going to be a pretty wide variation in some of this information, like proportion of visits where you prescribe an antimicrobial. Well, I'm a surgeon and so it's going to be a lot because I'm going to use intraoperative antimicrobials most of the time which is like its own soapbox as well as far as what's appropriate and what's not. Whereas, like our ICU clinicians, like it says critically important antimicrobial of the highest priority, I feel like our ICU clinicians are probably going to have a higher number of that than I am. And so should they necessarily be compared to me? I don't know. I mean, I don't know if it's right or wrong. I just legitimately don't. But I feel like that might be a little bit problematic just because it's probably not a fair comparison. But I feel like someone who specializes in micro infectious diseases would probably have to chime in on that to say if it truly is fair or not. -*Small Animal Surgery Resident*
**Suggestions for Improving the Report**	
ADD metric confusing	**Q7**. I think the ADDs of antimicrobial use per 1,000 animal days is probably not as helpful as the percentage of visits prescribed and breaking out what we might consider critically important drugs out of the field as defined by WHO maybe. I think—so I think that's probably a good starting point. I think—I just think the ADDs per 1,000 animal days I don't know. That's a tough one I think for people to get their head around potentially. Yeah. I mean I think these without benchmarks are not going to mean a lot to our docs. -*Small Animal Dermatology Faculty* **Q8**. The animal daily dose doesn't make a ton of sense to me, like I feel like I need to like really stop and read the sentences and think through them very slowly to actually understand what they're saying. But again, I am not a statistician. So, I'm sure to a much smarter person, this makes perfect sense. I am not one of those people. As far as calculating the animal daily dose, I don't know how that relates to clinical use. It seems complicated, but if this helps the researcher defining usage, great. I think I would need someone to—I can see the math, and if you gave me the numbers I could plug in the formulas and probably get the same numbers, but I'm not exactly sure what that tells us. *-Small Animal Surgery Resident*
Favorable perception of proportion and ranking metrics	**Q9**. Well, I think this is really interesting, just to see what classes we're prescribing the most, even though I figured sulfas would be—I thought penicillins would be more. This one's just more interesting than anything else. *-Large Animal Internal Medicine Resident*
Report contains too much information	**Q10**. I definitely think you can overwhelm people with data and I don't know that I would be wanting to give everybody each of these report types every month because their eyes are going to gloss over, they're going to open the email and go, “Oh that,” minimize, forget about it. But you know I think quarterly or biannually would probably be more palatable and then I think it's important at least once a year or so to have a grand rounds or a morbidity and mortality rounds to discuss how as a hospital things are looking, but how do you benchmark that is the question. *-Small Animal Dermatology Faculty*
Desire for benchmarks	**Q11**. There would have to also be, I think, an effort to standardize some guidelines or something. So just data and how you compare without any kind of gold standard or evidence-based recommendation is challenging to implement any changes. -*Large Animal Internal Medicine Faculty*
Desire for breakdown of data by case type	**Q12**. Have it broken down by the case type. So like the ten doctors here, for every laceration, you have the breakdown of what the prescribing doctors are using, or how long they're using them for this particular injury. And then that's where you can—that's how you potentially change the minds of someone that's overusing or misusing the antimicrobials. They're like, “Oh, shoot. For a simple laceration, either people are using only 3 days of antimicrobials, or nobody's using any. So why am I using 14 days of TMS, whereas everyone else is only using 5 days?” And that's where they're like, “Oh, geez. Maybe I'm not using it correctly.” *-Large Animal Surgery Resident*
Need comparison to standard of care for report to be behaviorally motivating	**Q13**. I think kind of like how are you measuring up vs. standard of care, that kind of information would be very helpful. I don't know that by itself, like just having this data here, don't know that it would necessarily—like this packet for me right at this minute won't necessarily change we I do in terms how I'm prescribing but, you know, if there was a little bit more information about like, you know, within the surgery department this is how many orthopedic cases you've cuts, this is how many that have gotten infected, this is how many that have gotten perioperative, plus or minus post-operative antimicrobials but, you know, I think that's just a lot of work for anybody to do. It's basically like a whole retrospective study. *-Small Animal Surgery Resident*
Accompany report with education and institutional clarity about goals	**Q14**. It would maybe just have it be a chance for Microbiology to sort of reflect on any new guidelines that they're recommending, especially as we sort of have some more. Because I think, over the next couple of years, there will be more formal guidelines, and there will be more evidence based medicine. So, to have both reflection and education about what we want people to change and focus on. Like— “hey, the hospital as a whole is using an awful lot of fluoroquinolones. Here are situations where maybe we should stop using that or switch to something else.” -*Small Animal Oncology Resident*
Clearly communicate non-punitive rationale behind report	**Q15**. And certainly, this would have to be preceded by explanations about the ethos and the—why this is being done and the remit behind it so that everyone's on board with the fact that, even if they find illustration of their metrics in this way a little bit aggressive, at least they know it's motivated by good. *-Small Animal Internal Medicine Faculty*
**Impact of Report on Prescribing Behavior**	
Report would change prescribing behavior	**Q16**. Yeah, absolutely, this would change how I use antimicrobials. I do think that—you know, we all learn in different ways, and I think this—giving us a visual representation of how we compare, I think that really does impact people a lot. Especially for people who are stuck in their ways, for sure. And I think that it does allow me to think of, “Does this patient really need an antimicrobial?” and will lead me to be a little bit more judicious when I'm choosing my antimicrobial therapy in the future. -*Small Animal Internal Medicine Faculty*
Report would not change prescribing behavior	**Q17**. I don't think this would change my prescribing. I hate to say it. I think I am actually somebody that is cognizant of antimicrobial resistance; it's something I do think about, and I know that we use a lot of antimicrobials, so this is not surprising to me. *-Small Animal Surgery Resident*
More granular data needed to change behavior	**Q18**. I don't know if it would impact things. I mean, even something more specific like orthopedics vs. airways, urogenital surgery; that's really, really detailed. It would probably be like a ton of work. But surgery is just such a broad title for what we do. Sometimes we're doing surgery in very contaminated places that the animal would be dead if you didn't put it on antimicrobials. And sometimes you're doing surgery, you just don't need it. -*Large Animal Surgery Resident*
Behavior change depends on performance and comparator	**Q19**. I think it would depend on the distribution maybe. So, like, finding myself on the 60 or 70th percentile as an intern probably wouldn't bother me vs. if I was like, “Oh, I'm on the 95th percentile compared to other internists on the East Coast.” Then yeah, that would definitely make me be like, “Oh, I'm definitely overprescribing this, that, or the other.” Or maybe for the duration of things, I'm going out longer than others. So, yeah, I think it could be potentially beneficial, but I think it would boil down to how it was applied and who you're comparing people to. *-Small Animal Internal Medicine Resident*
Would make excuses to justify poor performance	**Q20**. No, I would just say, for me personally. I would just say oh, it's my case population. Which might be wrong. Probably would be wrong. But that's what I would say. Unless all of my medicine friends looked very different from me. But then I would still say well, I'm a resident. I get the sickest cases. I would make excuses. *-Large Animal Internal Medicine Resident*

After considering the data in the reports carefully, all respondents expressed appreciation for the metrics. Many said they had not ever seen data like this before and felt, in general, that communicating any data about antimicrobial use could be an important technique to encourage veterinarians to think about their prescribing decisions in the aggregate, which could improve antimicrobial use (Q4). Respondents' critical feedback of the reports primarily focused on doubt that the data could account for the nuances of prescribing in diverse clinical scenarios that might cause some veterinarians to justifiably use more antimicrobials than their peers (Q5). In reflecting on how it felt to be compared to colleagues, respondents generally found this approach to be informative and motivating for behavior change. However, some expressed concern that the comparisons might not be fair based on each individual veterinarian's case mix (Q6).

### Specific Suggestions for Improving Report

While some respondents (*n* = 14, 41.2%) felt that the report was satisfactory and did not need changes, most had several suggestions as to how to improve the clarity and impact of the data. The majority of respondents (*n* = 22, 64.7%) found the animal defined daily doses metric to be the least meaningful and most confusing of all the metrics despite an explanatory page at the back of the report (Q7). Respondents suggested that this metric was not intuitive or clinically relevant and would take too much time for a non-statistically savvy, busy veterinarian to understand and find it meaningful (Q8). In comparison, respondents felt more favorably toward the proportion metrics and the ranking of antimicrobial classes (Q9). Some suggested moving the explanatory page that described how the metrics were calculated to the front of the report. Other respondents felt that the amount of information provided in the reports was overwhelming and suggested delivering less information all at once or using a phased approach so people could get used to receiving a report of this length (Q10).

One of the most common reactions to the report was a desire for benchmarks that could help put the individual's performance in context (Q11). We found, as respondents looked at the reports and thought aloud, that they felt the data would be more meaningful if it could be broken down further. Specific suggestions included organizing data by case or procedure type (Q12) and comparing the following: systemic vs. regional use of antimicrobials, colleagues only within the same specialty, prescribing data by species, farm vs. inpatient large animal use of antimicrobials, and prescribing data from other universities or hospitals.

The majority (*n* = 26, 76.5%) of respondents felt that supplementary information should be provided with reports to make them more actionable. For example, some respondents suggested that the metrics would only be meaningful and likely to produce behavioral change if it was clear that there was a “gold standard” or guideline that suggested what performance on each metric was “good” (Q13). Without a goal to strive for, many respondents expressed, the numbers alone would not motivate them to change. Others felt that the reports would be more useful if they were accompanied by institution-wide discussion about the trends and education about specific classes of antimicrobials that are overused or clinical scenarios in which prescribing could be improved (Q14). Most importantly, the “ethos” behind the report would need to be clearly communicated so prescribers would know that the intent of the report was to improve the quality of care delivered and not to punish individuals (Q15).

### Influence of Report on Prescribing Behavior

Respondents varied in whether they felt their prescribing behavior would change in response to receiving the reports. Some (*n* = 13, 38.2%) felt that the report would change how they prescribed antimicrobials (Q16). A smaller number (*n* = 8, 23.5%) felt that they are already making the most judicious prescribing choices possible and did not believe the report would change the way they prescribe (Q17). Others suggested that without a goal to strive for, the data were not motivating (Q13).

Other respondents (*n* = 13, 38.2%) were undecided as to whether the data would change their prescribing and offered a variety of reasons for this equivocation. They explained that prescribing choices are influenced by an individual's accumulated clinical experience and the circumstances of each case; therefore, it was difficult for them to imagine a report with such high-level, aggregate metrics prompting change in practice as a whole (Q18). These respondents found the data to be informative but felt that more detail would be needed before the reports would change how they use antimicrobials. Multiple individuals said that the impact of the reports would depend on where they found themselves on the distribution compared to their colleagues and whether they felt the comparison was fair (Q19). Other respondents were unsure if the reports would motivate them to change and admitted that they would likely think of “excuses” to justify their high-prescribing rates in comparison to their colleagues (Q20).

## Discussion

With increasing interest in antimicrobial stewardship in veterinary medicine (11, 28), methods to achieve stewardship goals are needed (29). In this study, we demonstrated the feasibility of providing individualized antimicrobial use reports to clinicians in veterinary hospitals, and we obtained feedback on the perceived utility of the reports in general and of the individual metrics used in the reports. The reports were generally well-received by clinicians, and all clinicians were appreciative of being able to visualize their antimicrobial prescribing patterns using different metrics.

Antimicrobial stewardship initiatives involving the provision of periodic feedback to clinicians on their antimicrobial prescribing have been used successfully in human medicine (6, 30–34) and in animal agriculture (7, 35, 36). In veterinary hospitals, such initiatives are being proposed (37, 38). However, as made clear by the participants of our study, who expressed a desire for information with which to gauge the appropriateness of their AMU, prescribing behavior should be benchmarked against universally accepted guidelines to be useful. In veterinary medicine, a general consensus about broad tenets of judicious AMU exist (39), but defined AMU guidelines exist for only a few select clinical conditions in small animal medicine (40–43). Adherence to these guidelines have been investigated within veterinary institutions (44, 45), but, to our knowledge, no use has been made of them to provide feedback to clinicians in a veterinary hospital setting. In small animals, antimicrobial use reports targeted to the clinical conditions for which guidelines exist represent the most logical option for a feedback-related antimicrobial stewardship intervention. Such interventions in the context of acute respiratory tract infections have been performed in human medicine (6, 33). However, targeted reports evaluate only a small proportion of an individual's prescriptions and may limit who can be evaluated, as veterinarians in certain specialties may not see patients with the conditions of interest. This is particularly problematic for large animal medicine, where AMU guidelines for specific conditions are lacking. The dearth of defined AMU guidelines represents an impediment to promoting appropriate antimicrobial use in the veterinary hospital, and more research is needed to develop such guidelines.

An important theme that emerged from our interviews was that of comparability. When benchmarking AMU across different units (e.g., clinicians, services, hospitals), comparability of patient populations seen by different clinicians is critical. Many of the veterinarians interviewed in this study expressed skepticism that their results could be meaningfully compared to those of their peers, as patient populations attended to differed greatly across and even within services. Situating an individual's prescribing patterns relative to peers within the same service is necessary but does not appear sufficient. Additional methods of ensuring comparability of patient populations seen by a clinician are needed. In human medicine, scoring systems such as the Medicare Severity Diagnosis Related Group or Charlson comorbidity score have been used when benchmarking AMU (46, 47). While scoring systems exist for specific conditions [e.g., equine colic (48, 49)], to our knowledge, there are no validated methods of more generally characterizing the disease severity or comorbidities of veterinary patients. Until such scoring systems are developed, clinicians may view attempts to compare their prescribing to peers' as invalid or unfair.

The question of which metrics to use when benchmarking or evaluating the appropriateness of AMU is a critical one that remains unresolved in both human medicine (50–52) and veterinary medicine (12, 53). In human medicine, a wide variety of AMU metrics exist for purposes of antimicrobial stewardship, many of which are similar to or equivalent to those used in our study (54). In animal agriculture, different metrics are used in different countries and systems (35, 55, 56), and an expansive body of literature has described and evaluated the advantages and disadvantages of these metrics (12, 23, 57–59). However, because herds of animals are treated on farms more often than individual animals, the metrics used on the farm setting may not translate well to the hospital setting. While there is some consensus among experts about which metrics should be used in the human hospital setting (60), others have argued that certain metrics, most notably the dose-based metrics which are equivalent to the ADD-based metrics used in this study, should not be used for purposes of antimicrobial stewardship (1, 52). This was corroborated by our study, as most of the clinicians found the ADD-based metrics to be confusing, even when provided with detailed explanations. While ADD-based metrics are appealing because they can be calculated from drug volumes that are often accessible in pharmacy or billing records, they are not intuitive. Moreover, we have shown that in veterinary medicine they are not, as they should theoretically be, equivalent to more intuitive duration-based metrics such as days of therapy (61) which have been used in human medicine for stewardship purposes (34). Dose-based metrics are useful for benchmarking AMU at the farm, regional, national, and international levels (62–67) and are generally recommended in these settings to monitor trends in use over time (12). Moreover, because the ADD represents a scaling factor more than an indicator of absolute AMU (23), it is well-suited for comparing a prescriber or user to his/her peers. However, at the level of the individual clinician working in a hospital setting, the ADD-based metrics are not immediately intuitive. It is unknown if prolonged exposure to these metrics will enhance a clinician's ease with these metrics, or if, as one clinician noted, they are just “tough […] for people to get their head around.” It has been suggested that duration-based metrics (e.g., individual days treated or number of individuals daily treated) may be more appropriate for hospital settings (12, 68). Unfortunately, these metrics were not easily accessible from our database and could not be extracted to present to clinicians.

In contrast, the proportion metrics (e.g., percent of visits where an antimicrobial or HP-CIA was prescribed) and the ranking of antimicrobial classes were the most well-received metrics. Both of these metrics or variants thereof [e.g., antimicrobial spectrum score (34, 69)] have been used successfully to gauge appropriateness of antimicrobial prescribing by physicians (6, 70) and veterinarians (44, 45, 71). These metrics capture information about the frequency and choice of antimicrobial prescriptions and can therefore be useful in encouraging clinicians to limit unnecessary prescribing of antimicrobials and optimize empiric antimicrobial regimens (e.g., targeted therapy over broad spectrum, lower vs. higher priority antimicrobials) (71, 72). Because they are also inherently intuitive, they were likely to be more readily accepted by clinicians.

Audit and feedback is a commonly used quality improvement intervention in human medicine. It has been demonstrated, however, that the effects of these interventions vary greatly and are not improving over time (13, 73). Simply providing professionals with data is not enough to stimulate change in the way they perform their work (74). Numbers must have salience and meaning to the individuals whose behavior is targeted for change. Antimicrobial stewardship feedback interventions that are consistent with the priorities, beliefs, and concerns of prescribers and that make meaningful social comparisons may be more successful than those that do not (5). Our study generates knowledge that can be used to inform the design and implementation of stewardship interventions in veterinary medicine. To advance the science of stewardship, interventions need to account for the social and behavioral mechanisms that are particular to the professional culture of veterinary medicine (75, 76).

Our study has several limitations. First, because we adopted a qualitative approach our findings may not be generalizable beyond the settings we studied. Second, despite explicit efforts to minimize their effects, our sample may be biased. It is possible that the respondents who agreed to participate to an interview possessed systematically different characteristics that influenced their willingness to participate and shaped their perceptions compared to those not interviewed. We were unable to assess the characteristics of respondents vs. non-respondents. It is also possible that interview respondents did not honestly share their perceptions about the Personalized Antimicrobial Use Reports in order to please the interviewer. Given that respondents did express criticism of the AMU metrics and their feelings about antimicrobial stewardship interventions, we believe the impact of social desirability bias on our data to be minimal.

## Conclusion

Providing feedback on antimicrobial prescribing to individual veterinary clinicians may be an effective antimicrobial stewardship intervention, as long as the metrics used to describe prescribing patterns are understood by clinicians. Prescribing frequency, durations of therapy, and ranking of antimicrobial classes appear to be the metrics most well-received by veterinary clinicians, while dose-based metrics associated with the ADD are less intuitive. However, more research is needed to establish antimicrobial use guidelines against which prescribing can be measured.

## Data Availability Statement

The raw data generated and analyzed during this study is not publicly available due to the sensitive nature of the data and ethics restrictions on data sharing. Respondents did not consent to have their data publicly shared. A de-identified dataset may be made available by reasonable request.

## Ethics Statement

The studies involving human participants were reviewed and approved by Institutional Review Board of the University of Pennsylvania. The patients/participants provided their written informed consent to participate in this study.

## Author Contributions

LR produced individual antimicrobial use reports for all clinicians, contributed to data analysis, and manuscript writing. BM interviewed participants and contributed to data coding and analysis. JS interviewed participants and contributed to data coding, data analysis, and manuscript writing. All authors contributed to the article and approved the submitted version.

## Conflict of Interest

The authors declare that the research was conducted in the absence of any commercial or financial relationships that could be construed as a potential conflict of interest.
