# Supplementary material for: Small and Large Animal Veterinarian Perceptions of Antimicrobial Use Metrics for Hospital-Based Stewardship in the United States
Source: Front Vet Sci. 2020 Sep 8;7:582. doi: 10.3389/fvets.2020.00582 (PMC7505943; doi:10.3389/fvets.2020.00582)
Supplement: Supplementary file 2 [file Table_1.DOCX]

**Interview Guide**

*Veterinarian Perceptions of Antimicrobial Stewardship*

**Demographics**

1. What is your job title?
2. What patient population do you see?
3. How long have you been working at this institution?
4. When did you finish veterinary school?

**Perceptions of Antimicrobial Resistance and Overuse in Veterinary Medicine**

1. What do you think about antimicrobial overuse in veterinary medicine?
2. Do you think your prescribing impacts antimicrobial resistance? [Please explain]
3. What are some reasons why a veterinarian might use antimicrobials when they are not needed?
4. How well do you think antimicrobials are used at your institution?
5. Can you think of any examples of inappropriate use of antimicrobials that you’ve seen in veterinary medicine?
6. Are there any antimicrobial use guidelines available to guide your practice? If yes, what are they? Do you use them?

**Perceptions of Antimicrobial Stewardship**

1. Antimicrobial stewardship programs have been implemented widely in human medicine. These programs are formally instituted and staffed by pharmacists and infectious diseases doctors. They contain a number of interventions intended to ensure the appropriate use of antimicrobials, including:
   1. Formulary restriction and prior authorization
   2. Prospective audit with feedback
   3. Antimicrobial “time outs”
   4. CDSS and electronic alerts prompting prescribers to consider changing therapy
   5. Benchmarking and prescriber report cards comparing individual to group performance
2. What are your thoughts about each of these elements in terms of implementation in veterinary medicine? The benefits? The drawbacks?

**Perceptions of the Personalized Antimicrobial Use Report**

[PRESENT RESPONDENT WITH REPORT]

1. What is your initial impression of what we just presented?
2. Does the report make sense to you? Does the metric?
   1. If not, how could it be made clearer?
3. How might you like to see these metrics and reports incorporated into your practice?
   1. How would you like them communicated to you?
4. Do you think these metrics and reports will impact how you use antibiotics? How come?
5. Do you have suggestions of how we can educate prescribers about these metrics and reports?
6. Is there anything else you'd like to add?
