# Supplementary material for: Small and Large Animal Veterinarian Perceptions of Antimicrobial Use Metrics for Hospital-Based Stewardship in the United States
Source: Front Vet Sci. 2020 Sep 8;7:582. doi: 10.3389/fvets.2020.00582 (PMC7505943; doi:10.3389/fvets.2020.00582)
Supplement: Supplementary file 1 [file Presentation_1.pdf]

Individualized antimicrobial use report

Dear Dr. [Name],

We are interested in your thoughts on the utility of tracking antimicrobial use at [Hospital name] using administrative databases and medical records. Many human hospitals with antimicrobial stewardship programs provide clinicians with individualized reports of their antimicrobial use patterns. We would like to know whether this is something you would potentially find helpful.

Antimicrobial use can be described using a variety of metrics.

We have produced a report of your antimicrobial prescribing over the past five years using the following metrics:

1) Percent of visits in which an antimicrobial was prescribed
2) Prescription rate (number of animal-defined daily doses* of antibiotics per 1,000 patient-days)
3) Average number of animal-defined daily doses* of antibiotics per patient
3) Average number of antibiotic types prescribed per visit
4) Most frequently prescribed classes of antibiotics

Your performance and that of other clinicians in the hospital is provided.
Each bar represents a different clinician. Yours is highlighted with a red oval

*The animal-defined daily dose roughly represents a day of antimicrobial therapy. More information on this metric with a worked example is provided at the end of this document.

1) <u>Proportion of visits where you prescribed an antibiotic:</u>

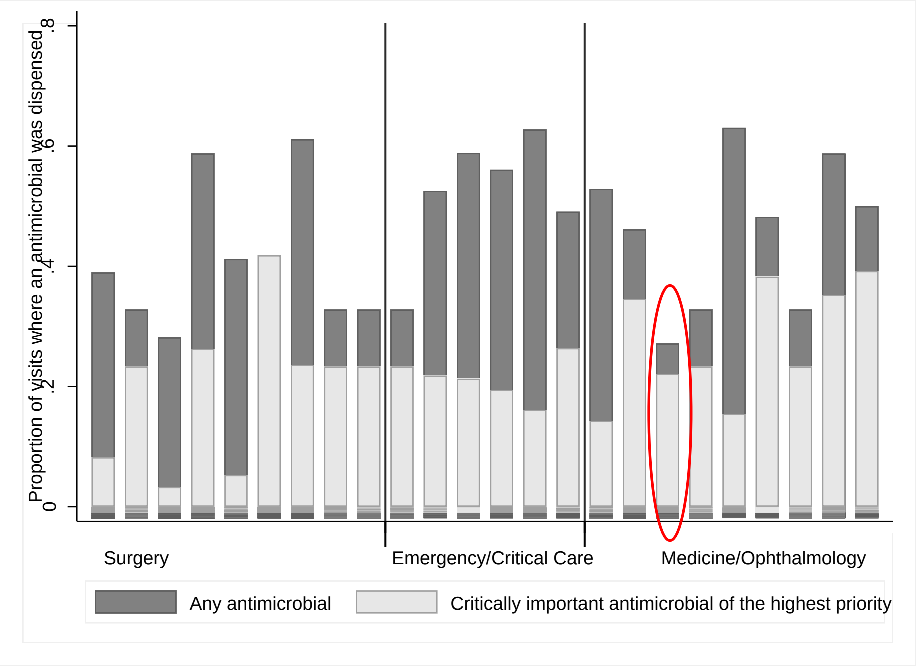

In the past five years, you prescribed antibiotics in 28% of visits

You prescribed a critically important antibiotic of the highest priority* in 22% of visits.

*3rd generation cephalosporins, glycopeptides, macrolides, ketolides, polymyxins, quinolones

2) <u>Prescribing rate – animal-defined daily doses per 1,000 patient-days</u>

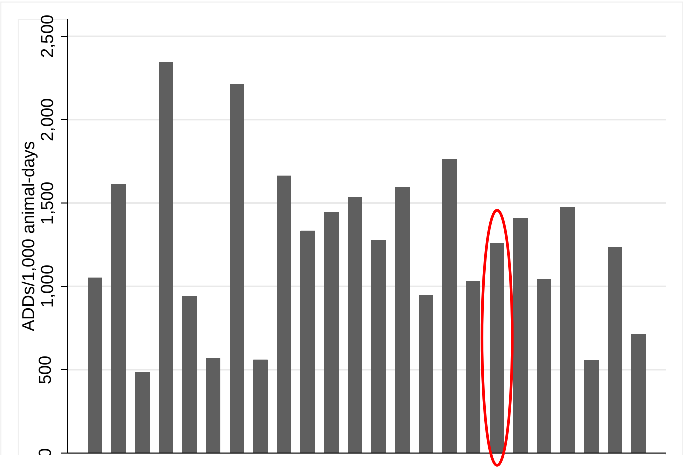

You prescribed antibiotics at a rate of 1,260 animal-defined daily doses per 1,000 patient-days in the previous year.

Note that this can happen (number of prescribed doses higher than the number of patient-days) when you prescribe individual animals more than one type of antibiotic and/or when you send animals home with antibiotics (and their length of treatment exceeds their hospital stay).

3) ADDs/patient

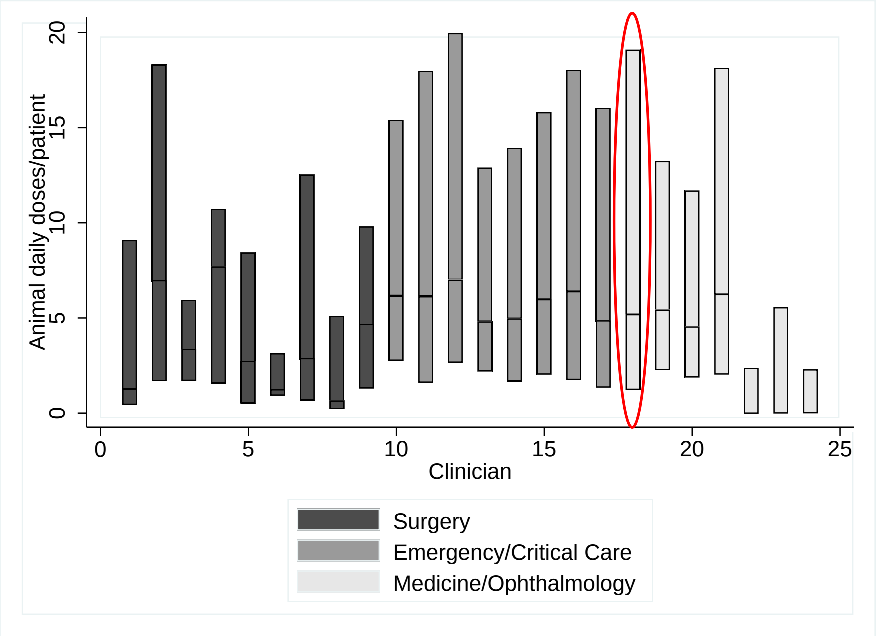

In this graph, the middle horizontal bar represents median values, while the bottom and the top of the rectangles represent 25th and 75th percentiles, respectively.

Your median number of ADDs/patient was 5.2 ADDs. This roughly corresponds to 5.2 days of antimicrobial therapy per patient.

4) <u>Antibiotic number – number of distinct classes of antibiotics prescribed per patient</u>

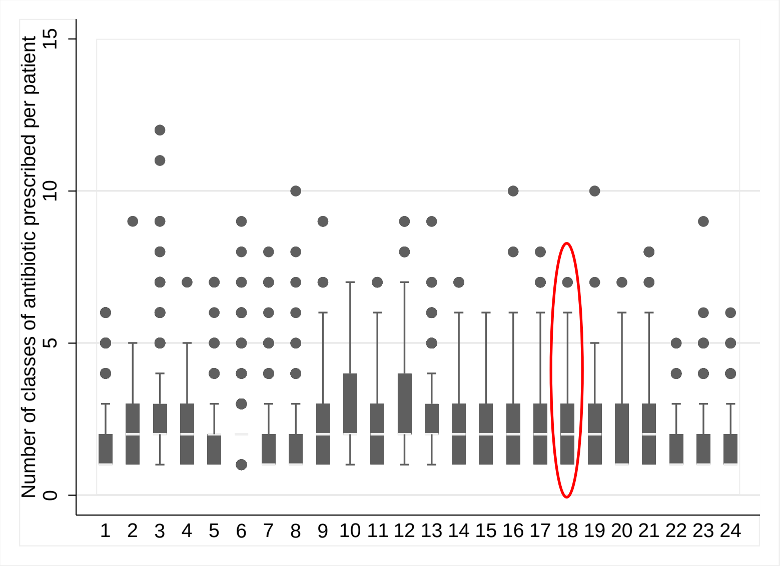

In this graph, the white horizontal bar represents the median value, while the top and bottom of the rectangles represent 25th and 75th percentiles, respectively. The dots represent outlier values greater than 1.5 times the 75th percentile (i.e., occurring infrequently).

On average, you prescribed 2 distinct classes of antibiotics per patient in the previous year

5) <u>Antibiotic rankings</u>

The following graph displays how often you prescribed different classes or combinations of classes of antibiotics.

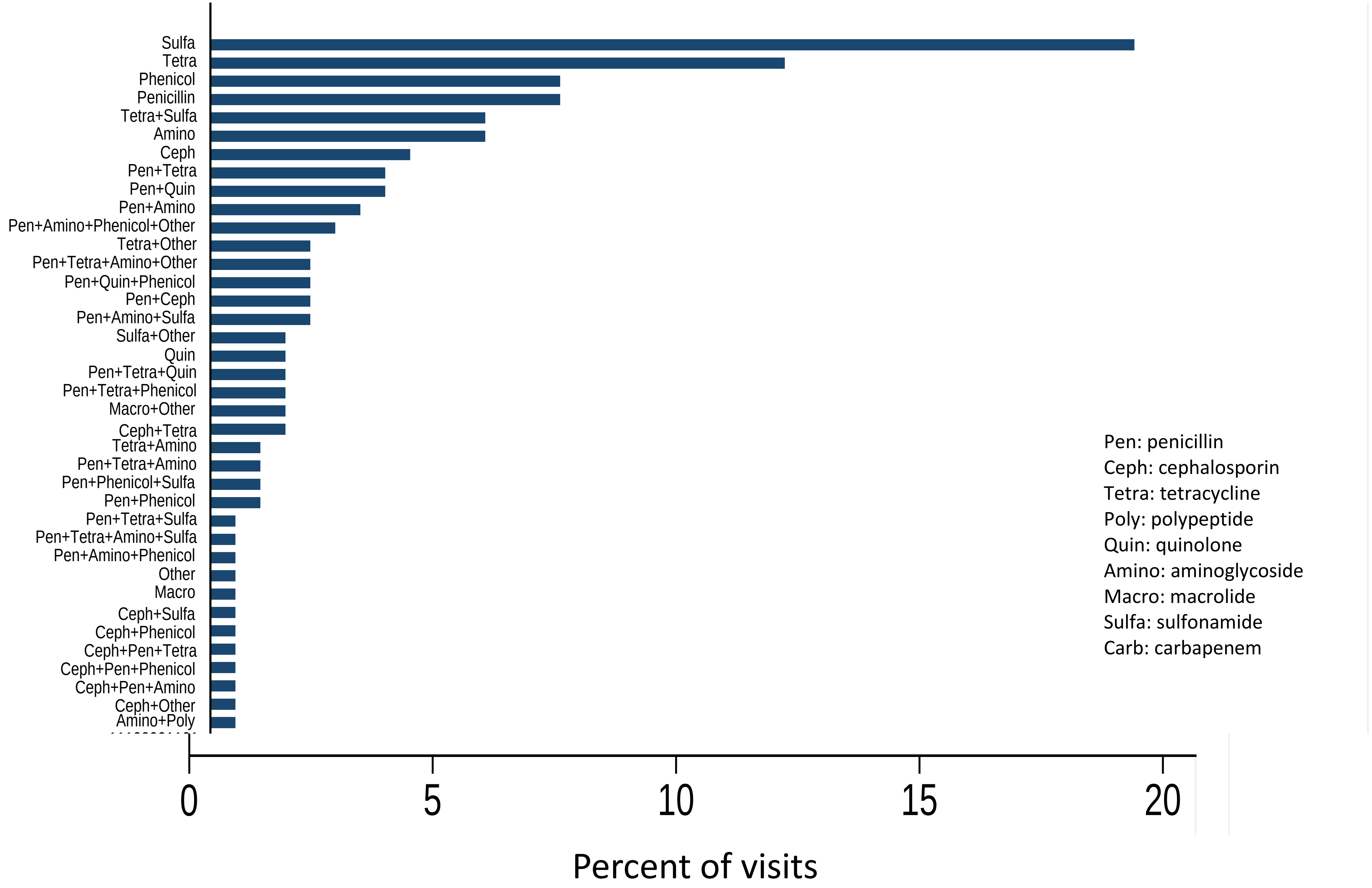

Animal Daily Dose:

The ADD (animal daily dose) is a unit of measurement that provides a rough estimate of consumption that is approximately equivalent to the number of days a patient was on one type of antibiotic.
The ADD/1,000 animal-days represents a rate of prescribing that is normalized to the number of patient-days seen by a clinician (as different clinicians can see different numbers of patients).

A worked example of how to calculate this metric is provided below.

Worked example for the calculation of animal-defined daily dose (ADD):

Horses are dosed at 22,000 i.u. of penicillin/kg QID.
A horse's **defined daily dose** (DDD_{vet}) of penicillin is therefore (22,000*4)=88,000 i.u./kg.

In one day, a 500-kg horse should receive 500 kg *88,000 i.u./kg=44,000,000 i.u.

A clinician admits a ~500-kg horse for a 24-hour stay. This horse was prescribed two 20-million-unit bottles of penicillin, or 40 million i.u.'s total.

If we divide the prescribed dose by the defined daily dose (i.e., what the horse actually received by what it should theoretically have received), we get the number of animal-defined daily doses:

$$\frac{Prescribed\ amount: 40\ million}{Defined\ daily\ dose: 44\ million\ units} = 0.9\ ADDs$$

This horse was also prescribed gentamicin.
Gentamicin is prescribed at a dose of 8.8 mg/kg SID, so a 500-kg horse should receive 500 kg x 8.8 mg/kg=4,400 mgs in one day.

This 500-kg horse was prescribed 50 ml's of gentamicin, or 50 ml x 100 mg/ml=5,000 mg's of gentamicin.

$$\frac{Prescribed\ amount: 5{,}000\ mgs}{Defined\ daily\ dose: 4{,}400\ mgs} = 1.1\ ADDs$$

This horse received **0.9 ADDs of penicillin and 1.1 ADDs of gentamicin, or 2.0 ADDs of antibiotic, over the course of one day**.
